# Supplementary material for: Improved Quality of Life Following Addiction Treatment Is Associated with Reductions in Substance Use
Source: J Clin Med. 2019 Sep 6;8(9):1407. doi: 10.3390/jcm8091407 (PMC6780566; doi:10.3390/jcm8091407)
Supplement: Supplementary file 1 [file jcm-08-01407-s001.zip › Pathways QOL Supp materials resubmit tracked changes.docx]

**Supplementary methods**

To determine the presence of a statistically-reliable reduction in number of days of use of the primary drug of concern (PDOC), reliable change criteria (RCC) for days of use of the PDOC were calculated using the Jacobson & Truax (1991) formula. This formula uses the reliability of a measure and the SD of the distribution of its scores at baseline to calculate the magnitude of change in an individual’s score that would be less than 5% likely to occur by chance in the absence of real change in the underlying construct. For reliability, we used the intra-class correlation (ICC) values reported by Ryan et al. (2014) for inter-rater reliability of the near-identical “days of use” measures from the Australian Treatment Outcomes Profile (alcohol 0.90; opioids 0.68; amphetamine-type substances 0.77, and cannabis 0.89). PDOCs that were reported by too few participants in our sample to calculate a reliable SD in days of use at baseline (cocaine, benzodiazepines), or for which Ryan et al. (2014) did not report ICCs (tobacco, ecstasy, GHB, inhalants, and synthetic cannabinoids) were grouped with pharmacologically similar PDOCs where applicable (i.e. cocaine and ecstasy grouped with meth/amphetamine as “amphetamine-type substances”; synthetic cannabinoids with cannabis) and, for the remaining PDOCs (benzodiazepines, tobacco, GHB, and inhalants), reliability was estimated as the mean of the reliabilities of the 4 main drug categories (0.81) and a pooled SD of baseline days of use for these participants was used to calculate RCC. RCCs for reduced frequency of use of the PDOC were 10, 12, 13, 18, and 18 days for those with alcohol, cannabis, amphetamine-type substances, opioids, and other substances, respectively, as their PDOC.

**Supplementary results**

In our main analyses, we grouped participants according to whether or not they showed “treatment response”, i.e. *either* abstaining from their PDOC in the month prior to follow-up, *or* showing a reliable reduction in number of days use of the PDOC in the month prior to follow-up, compared to the month prior to baseline. Thus, “treatment responders” included a mixture of participants who were abstinent from their PDOC at follow-up and those who were not abstinent, but who had substantially reduced how often they used their PDOC. To test whether there were differences between these sub-groups in changes in quality of life (QOL) between baseline and follow-up, we conducted supplementary repeated measures analyses of variance (RM-ANOVA) for each QOL domain with the sample divided into 3 groups, based on their PDOC use at follow-up: abstinent (n=206), non-abstinent treatment responders (n=72) and treatment non-responders (n=258). These data are shown in Figure S1.

Figure S1. Scores on each WHOQOL-BREF domain at baseline and follow-up among those who were abstinent at follow-up; those who reliably reduced use of their primary drug of concern (PDOC), but were not abstinent at follow-up; and those who did not reliably reduce the frequency of use of their PDOC.

When including all 3 groups in the analyses, significant interactions between outcome group (abstinent, non-abstinent treatment responders, and treatment non-responders) and time (baseline vs. follow-up) were found for all four QOL domains (physical: F(2,533)=25.578, p<.001, η^2^_p_=.088; psychological: F(2,533)=19.706, p<.001, η^2^_p_=.069; social: F(2,532)=13.184, p<.001, η^2^_p_=.047; environmental: F(2,532)=9.562, p<.001, η^2^_p_=.035). However, when analyses were confined to the two subgroups of treatment responders (i.e. excluding treatment non-responders), these interactions were absent (physical: F(1,276)=0.126, p=.723, η^2^_p_<.001; psychological: F(1,276)=0.40, p=.842, η^2^_p_<.001; social: F(1,276)=0.274, p=.601, η^2^_p_<.001; environmental: F(1,276)=1.522, p=.218, η^2^_p_=.005). Main effects of group were significant in these comparisons of the abstinent and non-abstinent treatment responders for all domains except social QOL (physical: F(1,276)=17.450, p<.001, η^2^_p_=.059; psychological: F(1,276)=17.318, p<.001, η^2^_p_=.059; social: F(1,276)=3.685, p=.056, η^2^_p_=.013; environmental: F(1,276)=7.140, p=.008, η^2^_p_=.025) because, as can be seen in Figure S1, QOL was generally lower in non-abstinent treatment responders than in abstinent treatment responders at both baseline and follow-up. However, the absence of any interaction with time suggests that both sub-groups of responders’ QOL improved to a similar extent between baseline and follow-up. Moreover, when participants who were abstinent at follow-up were excluded from these analyses, for the purpose of comparing non-abstinent responders to non-responders, interactions between time and group were significant for all domains except environmental QOL (physical: F(1,328)=20.480, p<.001, η^2^_p_=.059; psychological: F(1,328)=16.095, p<.001, η^2^_p_=.047; social: F(1,327)=9.384, p=.002, η^2^_p_=.028; environmental: F(1,327)=3.244, p=.073, η^2^_p_=.010), showing that non-abstinent responders’ QOL improved to a greater extent than non-responders on most domains.

Additional analyses were conducted to test whether the main finding (that treatment success was associated with improved QOL) was moderated by PDOC, particularly whether the strength of this association differed between those with alcohol as their PDOC and those with other PDOCs. To this end, RM-ANOVAs were conducted with time (baseline vs. follow-up) as a within-subjects factor and both PDOC (alcohol vs. all other drugs) and treatment success (responders vs. non-responders) as between-groups factors, to test for 3-way interactions between PDOC, treatment success, and time. These interactions were non-significant for physical (F(1,532)=1.086, p=.298, η^2^_p_=.002), social (F(1,531)=1.011, p=.315, η^2^_p_=.002) and environmental (F(1,531)=0.011, p=.918, η^2^_p_<.001). However, for psychological QOL, the interaction was significant, despite its effect size being small (F(1,532)=4.874, p=.028, η^2^_p_=.009). Figure S2 depicts this interaction, suggesting that the strength of the time x treatment response interaction was stronger among participants with alcohol as their PDOC than among participants with other PDOCs.

Figure S2. Changes in psychological QOL between baseline and follow-up among treatment responders (solid lines) and non-responders (dashed lines), shown separately for those with alcohol as PDOC (top panel), and those with any other drug as their PDOC (bottom panel).

To further explore this 3-way interaction, the strength of the 2-way interaction between time and treatment response was examined separately in the two PDOC groups. In those with alcohol as their PDOC, this interaction had a moderate-large effect size (F(1,266)=36.536, p<.001, η^2^_p_=.121). In those with non-alcohol PDOCs, the effect size of this interaction was substantially smaller, but still significant (F(1,266)=8.979, p=.003, η^2^_p_=.033). Thus, in both groups, treatment success was significantly associated with improved psychological QOL, but this effect was stronger among those with alcohol as their PDOC than among the rest of the participants. This could be consistent with either (or both) of two alternative explanations. Firstly, alcohol may be more harmful to psychological wellbeing than other drugs, and reducing or ceasing its use therefore produces more robust increases in psychological well-being. Secondly, alcohol use may be more difficult to reduce or cease in the context of poor psychological wellbeing than other drug use, perhaps due to ways in which alcohol is used to “self-medicate” psychological problems (e.g. anxiety).

We also explored 2-way interactions between PDOC and time to examine whether there were general differences between PDOC groups (irrespective of treatment success) in change over time in QOL. However, none of these interactions were significant (physical: F(1,532)=0.433, p=.511, η^2^_p_<.001; psychological: F(1,532)=2.009, p=.157, η^2^_p_=.004; social: F(1,531)=0.070, p=.791, η^2^_p_<.001; environmental: F(1,531)=0.762, p=.395, η^2^_p_=.001). Thus, there is no indication that PDOC had a general effect on change over time in QOL. For analyses of main effects examining associations between PDOC and QOL at baseline, we refer readers to already-published data presented in Lubman et al. (2016).
