# Supplementary figures and images for: Improved Quality of Life Following Addiction Treatment Is Associated with Reductions in Substance Use
Source: J Clin Med. 2019 Sep 6;8(9):1407. doi: 10.3390/jcm8091407 (PMC6780566; doi:10.3390/jcm8091407)

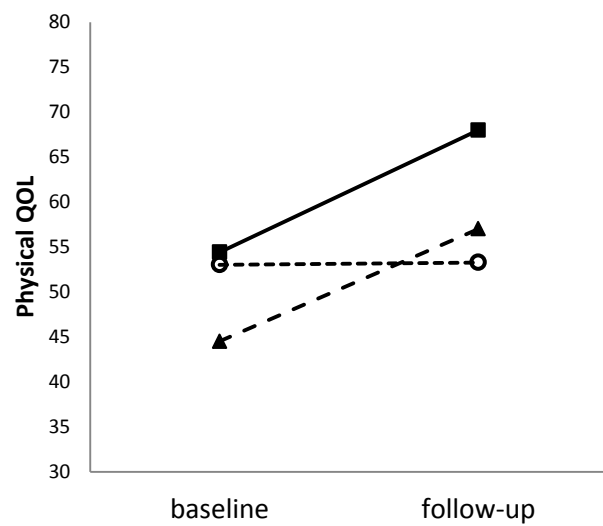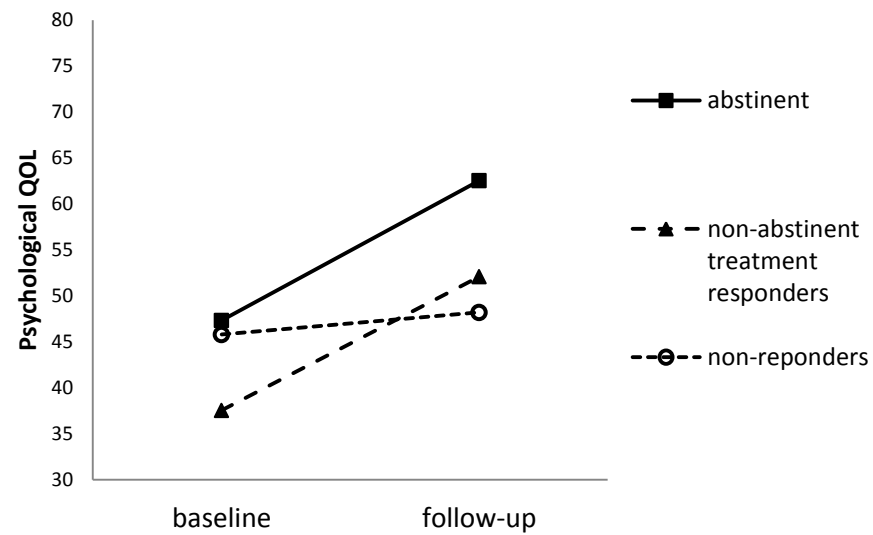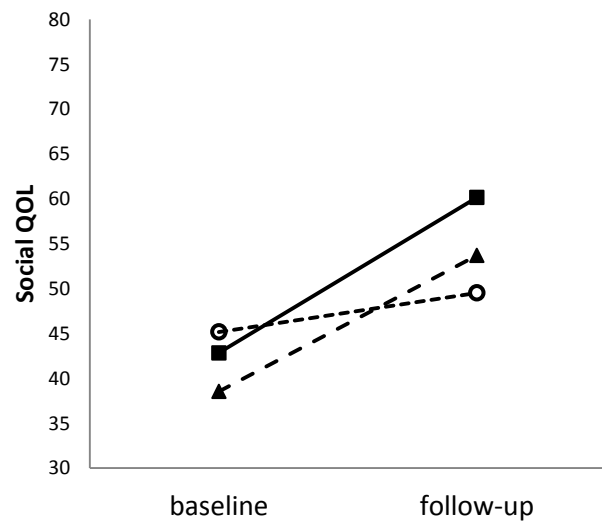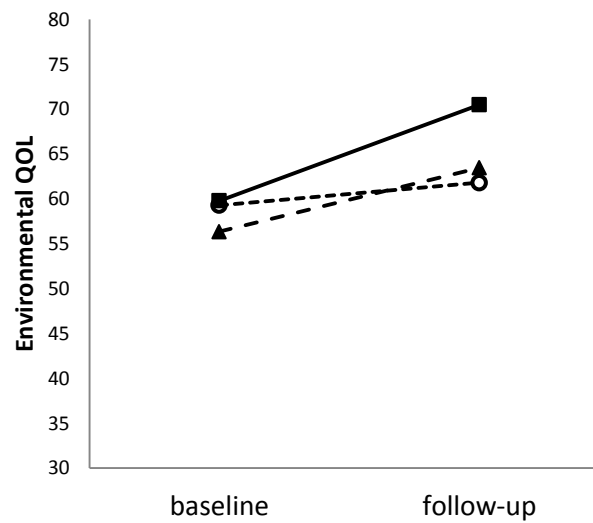

Supplement: Supplementary file 1 [file jcm-08-01407-s001.zip › Pathways QOL supplementary figure 1.pdf]

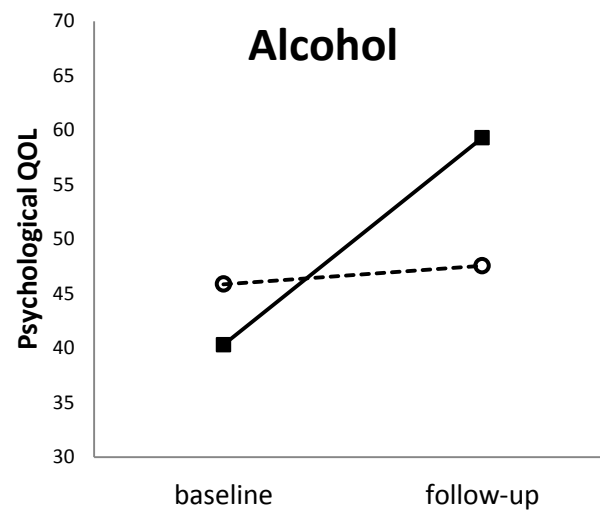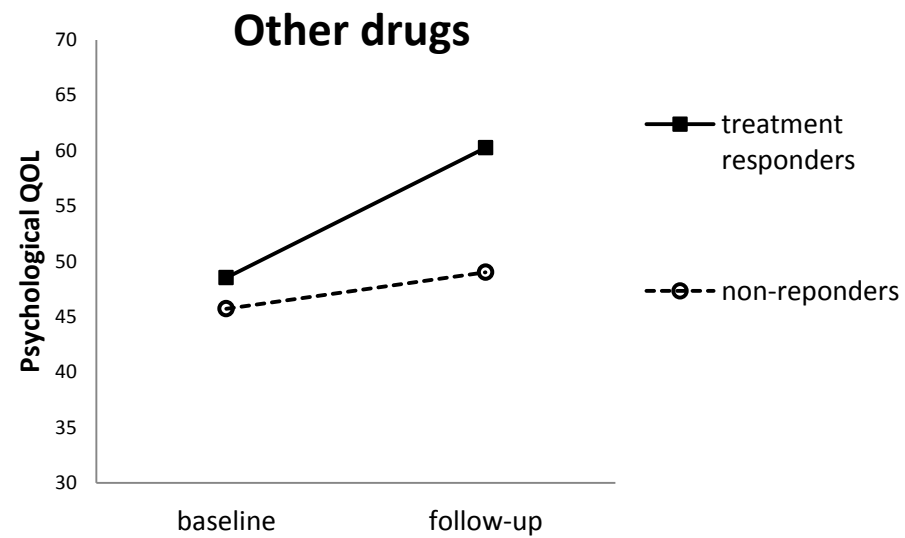

Supplement: Supplementary file 1 [file jcm-08-01407-s001.zip › Pathways QOL supplementary figure 2.pdf]
